# Operative Intervention for Lumbar Foraminal Gunshot Wounds: Case Report and Review of the Literature

**DOI:** 10.7759/cureus.5269

**Published:** 2019-07-29

**Authors:** Andrew Brash, Dia R Halalmeh, Gary Rajah, Joshua Loya, Marc Moisi

**Affiliations:** 1 Neurosurgery, Stony Brook University, New York, USA; 2 Neurosurgery, Detroit Medical Center, Detroit, USA; 3 Neurosurgery, Wayne State University School of Medicine, Detroit, USA; 4 Neurosurgery, Seattle Science Foundation, Seattle, USA

**Keywords:** lumbar spine, bullet, treatment, gunshot wound

## Abstract

Gunshot wounds represent the second most frequent cause of spinal cord injury after vehicular trauma. The thoracic region is most commonly involved, followed by the thoracolumbar spine. Numerous studies have demonstrated that improvement of neurological recovery, especially after decompression surgery, is likely to be seen in lumbosacral spine, but not in the thoracic or cervical spine. Herein, we present a case of a gunshot wound causing lumbar 5^th^ nerve root compression with neurological deficits that improved remarkably after urgent decompression surgery. This signifies a potential neurological benefit to prompt surgical intervention in lumbar gunshot wounds with radiographic evidence of neural compression. A relevant review of the literature was performed along with discussion, the clinical history, and radiological findings.

## Introduction

Violence represents the second most common etiology of spinal cord injury (SCI), only next to vehicular accidents [1]. Civilian spinal gunshot wounds (GSWs) have become increasingly common injuries in urban medical centers [2]. GSW to the spine accounts for 13-17% of all spinal trauma, more frequently sustained by young, male minorities between the ages of 15 and 34 years old [3,4]. In a systematic review of 1055 patients who sustained a civilian GSW, the estimated incidence of SCI at the cervical, thoracic, and the lumbosacral level was 30%, 49%, and 21%, respectively [3]. In the lumbar spine, the most frequent neurological injury is at L1, and the most common impairment is incomplete motor function (American Spinal Injury Association (ASIA) grade D) [1]. Specifically, the rate of incomplete SCI in GSW of the lumbosacral area is about 70% [5].

While there is a significant risk of complete SCI with thoracic GSWs, other factors such as race, missile trajectory, and the presence of bullet fragments in the spinal canal are significant predictors of neurological injury, specifically in regards to the lumbar spine [2,6]. Although evidence suggests that spinal fractures due to GSW tend to be stable and may not necessitate surgical intervention, some researchers advocate surgery due to possible intrathecal migration of the bullet, especially when close to the conus [7]. Moreover, the bullet composition can also be an appropriate indication for surgery. In one animal model study, the canine intervertebral disc was more susceptible to severe degenerative reaction upon exposure to copper alloys than the lead or aluminum alloys [8]. Furthermore, in the lumbar spine, there is potential for acute and chronic spinal instability if the bullet passes transversely, fracturing both pedicles and facet joints [9]. Foraminal bullets represent a specific subset of GSW to the spine, which in the lumbar region can have direct nerve root injury vs. causing dysfunction related to mass effect or blast effect.

Here, we report a 17-year-old female with GSW to the L5 neural foramen with corresponding neurological deficit. This article reviews the literature related to GSW-induced SCI and discusses the potential benefits for prompt surgical intervention in these injuries.

## Case presentation

**Figure 1:**
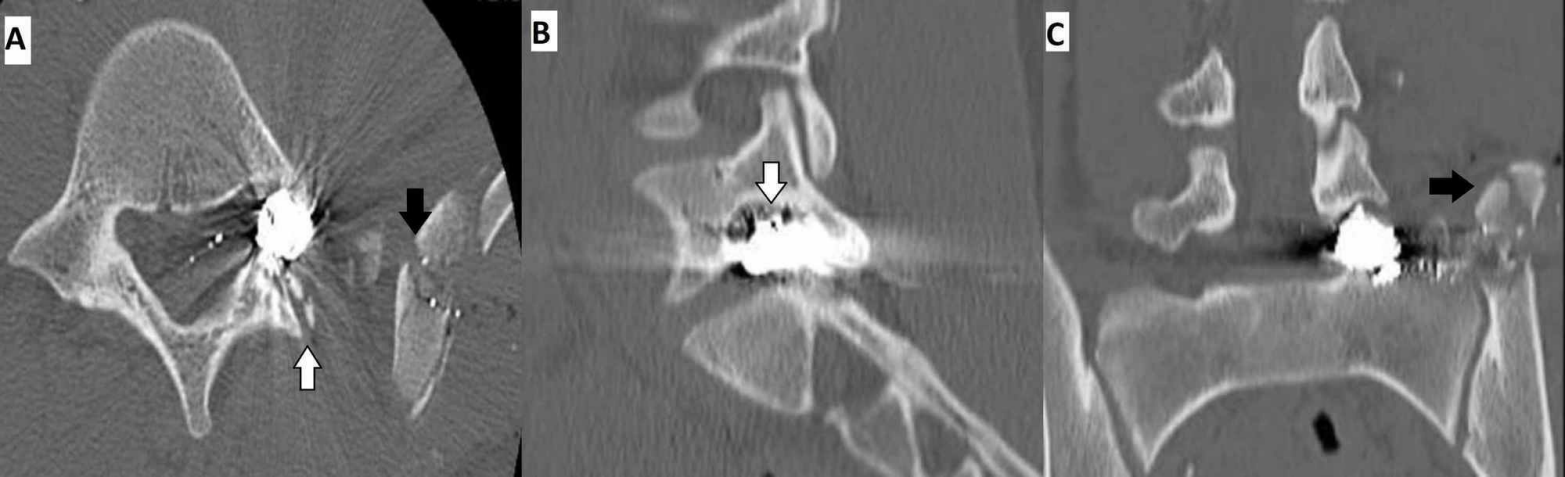
Preoperative CT scans demonstrating a high-density foreign body in the left articular facet of L5 neural foramen (A, B) axial and sagittal aspects showed fracture of the left L5 inferior facet (white arrows). No loss of vertebral body height or alignment. (A, C) axial and coronal aspects revealed comminuted blast fracture of the medial wing of the left iliac crest just superior to the left sacroiliac joint (black arrows), with no apparent joint destruction.

A 17-year-old female with no significant medical history presented to the emergency department after sustaining a gunshot injury to the left hip while exiting a vehicle. On initial examination, she was found to have 4/5 power in her left tibialis anterior (TA) and extensor hallucis longus (EHL) as well as decreased sensation to light touch in the L5 dermatomal distribution with an otherwise intact examination. A small entry wound on the left lateral flank at the level of the iliac crest was observed. CT of the lumbar spine revealed a comminuted fracture of the left iliac crest with a bullet lodged in the left L5 neural foramen associated with a comminuted fracture of the left S1 superior articular facet, suggestive of a trajectory through the iliac bone in a superior-medial direction (Figure 1). Bony spinal elements appeared otherwise grossly intact around the foreign body (FB).

**Figure 2:**
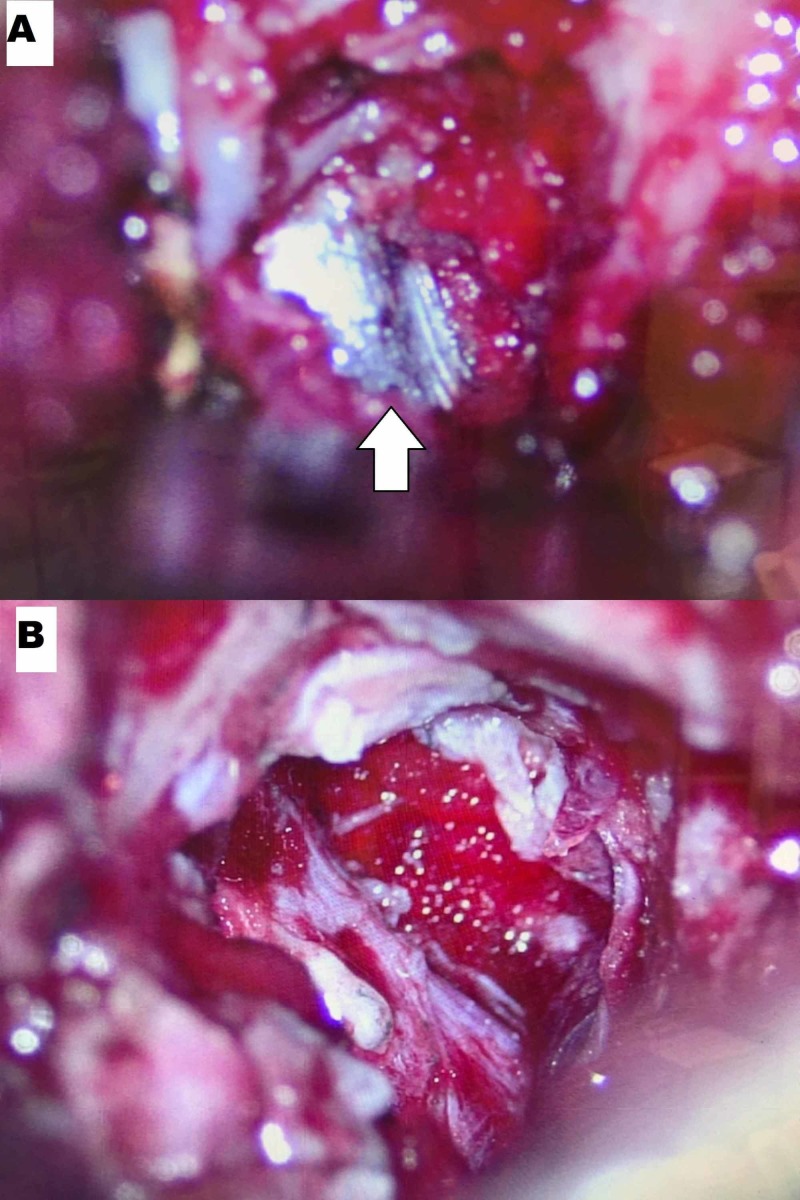
Intraoperative surgical view of the foreign body removal Surgical microscopic view before (A) and after (B) removal of the foreign body. Foreign body (white arrow)

**Figure 3:**
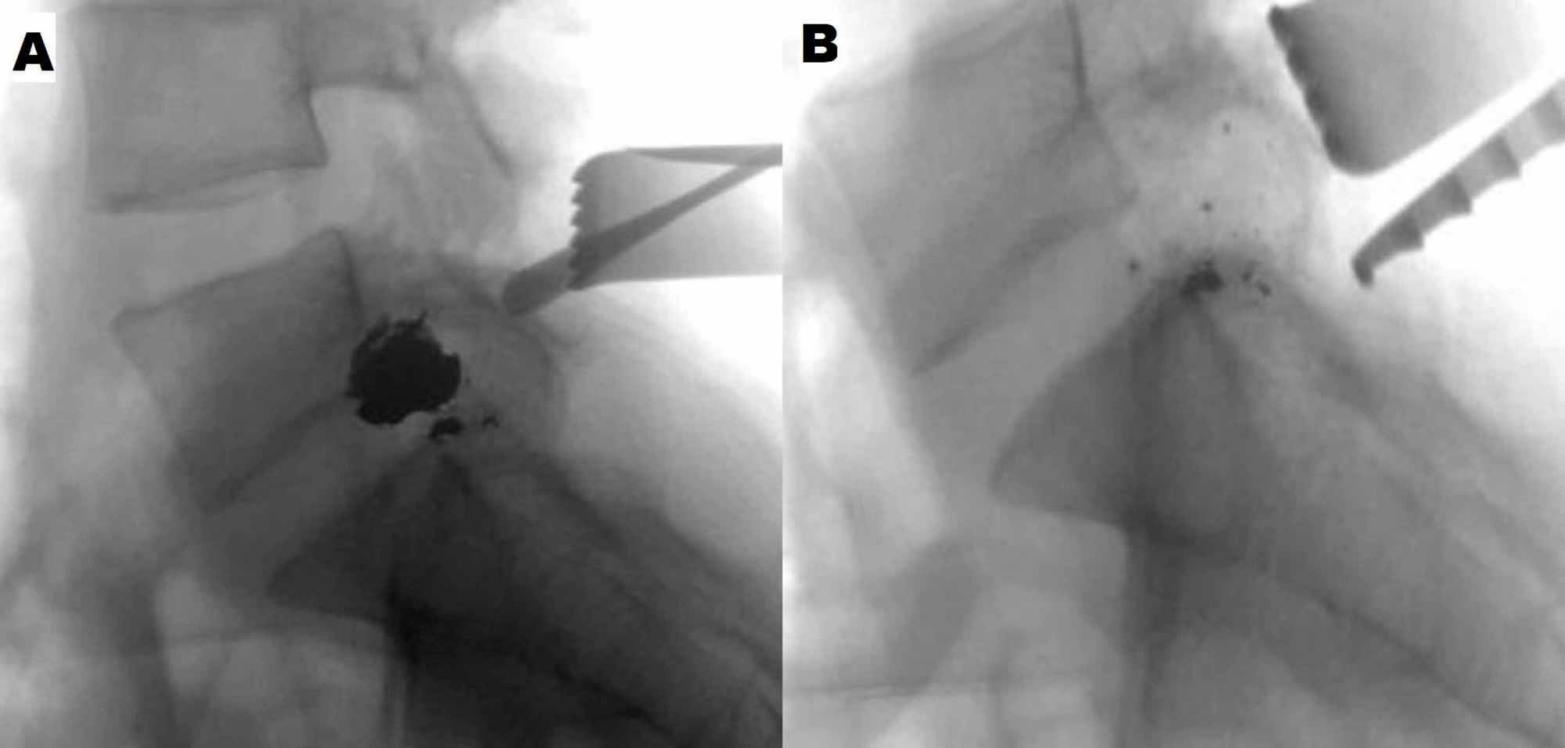
Intraoperative fluoroscopy demonstrating removal of the foreign body Intraoperative fluoroscopy before (A) and after (B) removal of the foreign body.

**Figure 4:**
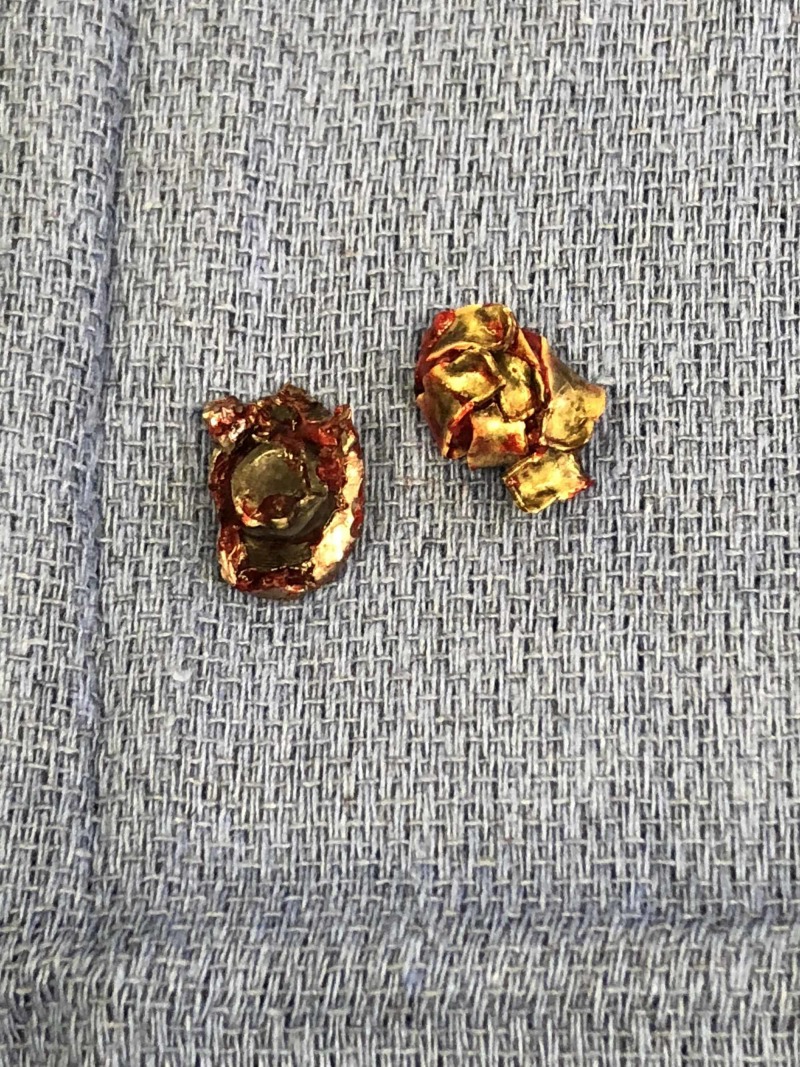
Foreign body

Given the patient’s new incomplete neurological deficit, attributable to active nerve root compression by the FB, the decision was made to take her to the operating room urgently for removal of the FB and decompression of the left L5 nerve root. The patient was positioned prone on a Jackson table and intraoperative fluoroscopy was used to localize the FB. A left L5 lateral laminotomy and L5-S1 facetectomy via a unilateral opening were performed, preserving the posterior tension band and contralateral muscle. The bullet was visualized wedged in the L5 neural foramen causing upward displacement of the left L5 nerve root (Figure 2A). The facetectomy was widened until the space was wide enough for the bullet to be dissected out of the foramen, taking precautions to avoid traction or trauma to the nerve root superiorly or the thecal sac medially. The dural sheath of the nerve root and the thecal sac were inspected and appeared intact without evidence of breach or cerebrospinal fluid (CSF) egress (Figure 2B). Intraoperative fluoroscopy confirmed complete removal of the major FB fragments (Figure 3). The FB was removed in two large intact pieces; a copper full metal jacket was noted superficially and the bullet was found deeper (Figure 4). After hemostasis was achieved, a Valsalva maneuver was performed without any evidence of CSF egress into the field. Post-operatively, the patient’s strength improved to 5 in TA and 4+ in EHL as well as improvement in L5 dermatomal sensation to near baseline.

## Discussion

Civilian GSWs are usually caused by low muzzle velocity (<1000 ft/sec) handguns, damaging tissues and structures by direct impact injury along the bullet’s path, pressure or shock waves created by the bullet impacting on tissue, and temporary cavitation [10]. The severity of gunshot injuries to the spinal cord depends on ballistics, degree of cord contusion and transection, degree of blast injury, compression of the cord by displaced bullet fragments or hematoma, disruption of the vasculature, and mechanical instability of spinal segments [5].

Upon approaching the patients with GSW of the spine, an initial evaluation must address stabilization, including airway, breathing, and circulation with consideration to the region of injury followed by a detailed neurological examination. After plain films have determined the level of injury, computed tomography is the advanced modality of choice for GSW of the spine as the use of MRI is controversial due to the risk of migration, especially in the acute settings [4]. 

In cases involving a bullet in the spinal canal, there is no clear consensus when to pursue surgical management versus a conservative approach involving pain management and rehabilitation. Some authors suggest decompressive laminectomy in the setting of incomplete neurologic deficits, with instrumentation and fusion if the injury is deemed mechanically unstable [10]. In addition, it has been proposed that removal of foreign objects carries a higher potential for axonal regeneration of injured nerve roots [7]. Operative management has been found to improve symptomatic partial nerve root injury in the lumbosacral spine, yet minimal benefit was observed at the cervical and thoracic levels [3,4]. Furthermore, a recent meta-analysis reviewed the outcomes of surgical management for SCI due to penetrating shrapnel as well as high and low-velocity bullets. While these have the potential for both direct nerve injury and blast injury of nearby neural structures, bone, and soft tissues, the authors found no clear benefit of decompression [11]. Although the literature consistently supports surgical intervention in the setting of post-gunshot spinal infection, persistent CSF leaks and documented progression of neurologic deficits warrant further study to understand which patients may benefit most from surgical intervention [2,4].

In our opinion, this patient’s new neurologic deficit, most likely attributable to a retained bullet in the L5 neural foramen with active compression of neural elements, was an indication for urgent surgical decompression. Minimally invasive surgery (MIS) of the spine has proven to be effective for herniations, discectomies, decompressions, and fusions with decreased tissue trauma, postoperative pain and narcotic use compared to traditional, open procedures [12-14]. More recently, MIS has been applied in the setting of trauma, specifically in the treatment of a sacral gunshot injury and for exposure of the neural foramen to safely remove a retained bullet from the spinal canal [15,16]. An open posterior approach has also been reported for foraminal bullet due to the presence of multiple fragments, thus requiring good exploration [17]. In our case, a unilateral open approach was chosen to focus on the unilateral pathology (compressive fragment) and to avoid injury to the posterior tension band and surrounding muscle. Although the operative view was reduced relative to the traditional open approach, identifying relative bullet position from pre-operative and intra-operative imaging allowed for successful extraction with minimal risk of nerve injury.

Foraminal bullets are space-occupying lesions that pose the risk of direct ongoing compressive nerve injury beyond the initial concussive or lacerating injury. The potential for multiple fragments and proximity to emerging nerve roots can make incidence and progression of neurologic deficit more likely. A common tenet of trauma neurosurgery is that surgical decompression is merited if active compression of neural elements is present and causing a new or progressive neurological deficit. As discussed above, GSW injuries to the spine can be multifactorial and include concussive, penetrating, and shearing trauma to the neural elements for which surgical intervention may be of limited utility in the absence of instability. However, this case demonstrates that active neural compression by retained bullet fragments must also be considered and evaluated in a patient with a new focal neurological deficit after a spinal GSW. Similar to any other mass lesion, the presence of a foraminal bullet and corresponding neurological deficit may warrant surgical exploration and removal of bullet fragments to optimize functional recovery. The case presented here illustrates a minimally invasive approach to neural decompression and fragment removal to be a viable treatment strategy for these select cases. 

## Conclusions

Although there may be no major improvement of neurological deficit after surgical intervention for GSW-induced SCI, early operative intervention might potentially improve outcomes in cases with radiological evidence of neural compression and progressive neurological deficit. Further research is required to identify potential outcomes of early operative intervention for SCI caused by GSWs.
